# Uncovering Novel Viral Innate Immune Evasion Strategies: What Has SARS-CoV-2 Taught Us?

**DOI:** 10.3389/fmicb.2022.844447

**Published:** 2022-03-23

**Authors:** Douglas Jie Wen Tay, Zhe Zhang Ryan Lew, Justin Jang Hann Chu, Kai Sen Tan

**Affiliations:** ^1^Biosafety Level 3 Core Facility, Yong Loo Lin School of Medicine, National University of Singapore, Singapore, Singapore; ^2^Department of Microbiology and Immunology, Yong Loo Lin School of Medicine, National University of Singapore, Singapore, Singapore; ^3^Infectious Disease Translational Research Programme, Yong Loo Lin School of Medicine, National University of Singapore, Singapore, Singapore; ^4^Department of Otolaryngology, Yong Loo Lin School of Medicine, National University of Singapore, Singapore, Singapore; ^5^Collaborative and Translation Unit for Hand, Foot and Mouth Disease (HFMD), Institute of Molecular and Cell Biology, Agency for Science, Technology and Research, Singapore, Singapore

**Keywords:** SARS-CoV-2, viral-host interactions, innate immune evasion, protein-protein interactions, RNA-protein interactions, RNA-RNA interactions

## Abstract

The ongoing SARS-CoV-2 pandemic has tested the capabilities of public health and scientific community. Since the dawn of the twenty-first century, viruses have caused several outbreaks, with coronaviruses being responsible for 2: SARS-CoV in 2007 and MERS-CoV in 2013. As the border between wildlife and the urban population continue to shrink, it is highly likely that zoonotic viruses may emerge more frequently. Furthermore, it has been shown repeatedly that these viruses are able to efficiently evade the innate immune system through various strategies. The strong and abundant antiviral innate immunity evasion strategies shown by SARS-CoV-2 has laid out shortcomings in our approach to quickly identify and modulate these mechanisms. It is thus imperative that there be a systematic framework for the study of the immune evasion strategies of these viruses, to guide development of therapeutics and curtail transmission. In this review, we first provide a brief overview of general viral evasion strategies against the innate immune system. Then, we utilize SARS-CoV-2 as a case study to highlight the methods used to identify the mechanisms of innate immune evasion, and pinpoint the shortcomings in the current paradigm with its focus on overexpression and protein-protein interactions. Finally, we provide a recommendation for future work to unravel viral innate immune evasion strategies and suitable methods to aid in the study of virus-host interactions. The insights provided from this review may then be applied to other viruses with outbreak potential to remain ahead in the arms race against viral diseases.

## Introduction

Seasonal strains of Influenza and periodic epi/pandemics, such as the latest SARS-CoV-2 pandemic are a grave concern for public health. With our developments encroaching into natural habitats, it is unavoidable that the barrier between reservoir hosts for viruses and the human population is slowly eroded, increasing risk of emerging zoonotic viruses (Murray and Daszak, 2013; Allen et al., 2017). This is evidenced circumstantially by the recent betacoronavirus epidemics; Severe Acute Respiratory Syndrome coronavirus (SARS-CoV; 2002) (Song et al., 2005), Middle East Respiratory Syndrome coronavirus (MERS-CoV; 2012) (Han et al., 2016) and SARS-CoV-2 (2019) (Andersen et al., 2020; Leitner and Kumar, 2020; Zhang and Holmes, 2020; Kadam et al., 2021). The latest pandemic caused by SARS-CoV-2 has demonstrated the widespread transmission and severity of highly pathogenic respiratory viruses, causing ∼5.7 million deaths (as of 3 February 2022) since its emergence.

When encountering a viral infection, the innate immune response (first line of antiviral response) efficiently inhibits viral replication through production of type I/III interferons (IFNs) and their respective interferon stimulated genes (ISGs). The unique molecular patterns of viruses are detected in host cells *via* various pattern recognition receptors (PRRs). For example, viral RNA is recognized by Toll-like receptors (e.g., TLR3, 7, and 8) and RIG-I-like receptors (RLRs), while foreign DNA is recognized by cytosolic DNA sensors [e.g., cyclic GMP-AMP synthase (cGAS)]. The activation of innate immune sensors triggers a signaling cascade ultimately leading to the induction of type I and/or type III IFNs. The type I IFN (consists mainly of IFNα/β) binds to IFNα receptors 1 and 2 (IFNAR1/2) and type III IFN (consists of IFNλ1, 2, and 3) binds to IFNλR/IL-10Rβ, activating expression of hundreds of ISGs to inhibit the viral replication cycle. Many studies have also shown the potency of IFN pretreatment prior to viral infection in inhibiting viral replication (Davidson et al., 2016; Lei et al., 2020; Hong et al., 2021). However, viruses have also evolved mechanisms to suppress and/or circumvent the innate host defenses as a response to maintain replicative fitness, usually to the detriment of the host. Since the emergence of SARS-CoV-2 causing the COVID-19 pandemic, it has been shown that the SARS-CoV-2 virus can very effectively subvert innate immune responses which indirectly contribute to disease severity in infected patients (Hatton et al., 2021). The delayed induction of IFN due to its suppression by viral evasion mechanisms is associated with viral persistence and inflammatory damage; often, cytokines are elevated in such scenarios, leading to cytokine storm and a worse prognosis (Park and Iwasaki, 2020; Sa Ribero et al., 2020; Ramasamy and Subbian, 2021). This has once again put in focus our limited understanding of the arsenal viruses possess to evade our innate immune responses and require urgent attention to be paid to it.

With the ever-looming threat of the next pandemic, understanding how viruses evade the immune response may allow us to devise novel antiviral treatments and strategies, or repurpose existing drugs as a rapid countermeasure to emerging threats; especially with a monumental case study such as the SARS-CoV-2. Furthermore, identifying the viral genes required for host immune suppression may aid in the development of a recombinant virus with key deletions as a vaccine candidate. Recent developments in methodologies have allowed for higher throughput analyses to be performed, providing new tools for *de novo* identification (e.g., next-generation sequencing technologies) and virus-host interaction studies (e.g., CRISPR gene knockout screens have been used to determine which gene products associate with the virus molecules).

In this review, we first provide a brief overview of general evasion strategies against the innate immune system. Then, we utilize SARS-CoV-2 as a case study to highlight the methods used to identify the mechanisms of innate immune evasion. Finally, we provide a recommendation for future work to unravel the innate immune evasion strategies by viruses, and additional methods to aid in the study of virus-host interactions. The insights provided from this review may then be applied to other viruses with outbreak potential to remain ahead in the arms race against viral diseases.

## General Viral Evasion Strategies of the Innate Immune Response and Their Discovery

Viral evasion of the innate immune system can be broadly summarized into a few categories: (a) interference of host pathogen sensors and antiviral factors, usually affecting the type I (IFNα/β) and/or type III (IFNλ) interferon signaling pathways, (b) modification of 5′-end of viral mRNA, and (c) formation of replication organelles. Some examples of common viruses are given, while SARS-CoV-2 will be covered in the next section. Methods used for identification of viral-host interactions in each study will be included in parenthesis **in bold**.

### Interference of Host Pathogen Sensors and Antiviral Factors

The viral genome consists of structural, non-structural, and accessory proteins. These proteins, and even viral RNA, can perturb the innate immune response in the host cell. Below are examples of each class of molecules interfering with the function of the innate immune system, leading to viral evasion.

#### Structural Proteins

SARS-CoV M protein has been found to interfere with formation of the TRAF3.TANK.TBK1/IKK_ε_ complex, thereby interfering with the activation of the type I IFNs (Siu et al., 2009) (**Co-Immunoprecipitation**). Influenza M1 protein has been found to interact with C1qA of the complement system, inhibiting its interaction with IgG, thereby protecting the Influenza virus from complement mediated neutralization *in vitro* (Zhang et al., 2009) (**GST and His pull-down, Yeast two-hybrid, Co-immunoprecipitation).**

#### Non-structural Proteins

Influenza NS1, perhaps a prime example of innate immune evasion (Ji et al., 2021), perturbs innate immunity in the following ways: (a) inhibiting the type I interferons through a variety of methods (including but not limited to interacting with the CARD domain of RIG-I) (Jureka et al., 2015), (b) inhibiting the general expression of genes by inhibiting the mRNA export complex (Satterly et al., 2007), and (c) directly interacting with ISGs (such as protein kinase R) to antagonize their effects (Min et al., 2007; **Immunoprecipitation,** Min et al., 2007; Satterly et al., 2007; **Nuclear Magnetic Resonance,** Jureka et al., 2015). ZIKV (Zika virus) NS4A interacts with the CARD domain of the mitochondrial antiviral signaling protein (MAVS), thereby interfering with the interaction between MAVS and RIG-I/MDA5, ultimately preventing the expression of type I IFNs (Ma et al., 2018) (**CRISPR screen**).

#### Accessory Proteins

MERS-CoV ORF8b interferes with the interaction of IKKϵ and HSP70-1A by competing with IKKϵ for HSP70-1A, preventing signaling through the IRF3 pathway from proceeding, thereby inhibiting the production of IFNβ (Wong et al., 2020) (**Immunoprecipitation-Mass Spectrometry [MS]**). Influenza A PB1-F2 has been shown to inhibit interferon induction downstream of the pattern recognition receptor pathway, RIG-I-TRIM25, by interacting with MAVS (Conenello Gina et al., 2011; Varga et al., 2011) (**Functional Screen**).

#### RNA

DENV (Dengue virus) subgenomic flavivirus RNA is known to interfere with the innate immune system through its direct interactions with TRIM25 and interfering with its deubiquitylation, thus interfering with the RIG-I signaling and consequently, type I IFN production (Manokaran et al., 2015) (**RNA-Immunoprecipitation**).

#### Viral Envelope/Envelope Proteins

Influenza has been known to incorporate CD59 into the membrane of the virion, allowing it to inhibit the formation of the membrane attack complex by the complement system (Shaw et al., 2008) (**Liquid Chromatography Tandem Mass Spectrophotometry, Immunogold Labeling**).

### Modification of 5′-End of Viral mRNA

RLRs are pattern recognition receptors in the cytoplasm that detect viral RNA. Host RNA molecules are modified with a 5′ cap that prevents RLRs from recognizing them as foreign RNA (Daffis et al., 2010). Viruses have evolved to appropriate these 5′ caps or mimic it to preserve viral RNA by evading innate immune detection. The mechanisms by which this takes place are unique to each virus, a few examples are listed for reference.

Influenza viruses have been known to engage in a process known as “cap snatching.” Viral RNA bound RNA-dependent RNA polymerase (RdRp) associates with the C terminal domain of RNA polymerase II, allowing interaction of polymerase basic protein 2 (of RdRp) with the 5′ cap of host RNA. Host RNA is then cleaved 10–13 nucleotides down, producing a capped oligonucleotide that is used to prime viral RNA transcription (Sikora et al., 2014; De Vlugt et al., 2018) (**Functional Screens**) (Bouloy et al., 1978; Plotch et al., 1979). The polymerase complex of coronaviruses, like SARS-CoV, have an inbuilt capping function. NSP14 of SARS-CoV acts as a guanine-N7-methyltransferase, a major component in the cap snatching process (Chen et al., 2009). NSP16 of SARS-CoV prevents detection of viral RNA by MDA5, an RLR, by further modification of the product of NSP14 through its 2′-O-methyl-transferase activity (Chen et al., 2011) (**Functional Screens**).

### Replication Organelles

Replication organelles, also known as double membrane vesicles (DMVs) and invaginations, are appropriated intracellular membranes that may, speculatively, shield viral PAMPs from the innate immune sensors present within the cytoplasm (Stertz et al., 2007; Miorin et al., 2012; Uchida et al., 2014). These structures are usually found in infections by positive strand RNA viruses, which may require this mechanism to prevent the foreign configuration of viral molecules formed during viral replication (e.g., double stranded RNA) from triggering the innate immune mechanisms that lead to their degradation.

DENV, for example, conceals its dsRNA within DMVs, reportedly diminishing the impact of the host innate immunity (as determined by IFNβ expression), as compared to Japanese encephalitis virus, whose dsRNA is detectable in the cytoplasm much earlier than DENV (Uchida et al., 2014) (**Immunofluorescence Assay, RT-qPCR**). It has, however, been shown that at least in coxsackievirus (an enterovirus), replication organelles do not seem to result in enhanced innate immune evasion (Melia et al., 2017) (**Localization *via* Electron Microscopy/Fluorescence Microscopy**). With diverse observations, the impact of replication organelles on innate immune evasion may be unique to each viral infection and mechanisms behind this phenomenon are still relatively unclear, requiring further research for each virus of interest.

## Identification of Viral Innate Immune Evasion Mechanisms: a SARS-CoV-2 Case Study

### Determining Whole Genome Sequence and Gene Expression

The current SARS-CoV-2 pandemic offers a suitable case study for the pipeline of elucidating viral evasion strategies in an emerging pathogen. In February 2020, the first genome of SARS-CoV-2 (Wu et al., 2020) was determined *via* metagenomic RNA sequencing, and multiple sequence alignment with known coronaviruses identified a similar order of genes to betacoronaviruses: 5′ -replicase (ORF1ab), spike (S), envelope (E), membrane (M), and nucleocapsid (N)-3′ genes. The ORF1ab polyprotein is comprised of 16 NSPs (Yoshimoto, 2020). Numerous studies have attempted to map the expression profile of SARS-CoV-2, though with conflicting results, especially regarding the number of functional accessory proteins. The current annotation (GenBank: NC_045512.2), based on predictions from sequence homology with other coronaviruses, consists of 6 accessory proteins (3a, 6, 7a, 7b, 8, and 10).

A study using direct RNA sequencing of SARS-CoV-2 infected cells found subgenomic RNAs for 5 of the accessory proteins (3a, 6, 7a, 7b, and 8), but ORF10 was not detected (Kim et al., 2020). Another study looking at the translatome of SARS-CoV-2 using ribosome profiling showed that the proteins annotated in the NCBI reference genome were expressed (except ORF10, though translation initiation signal was suggested to be present) and identified 23 unannotated viral ORFs, with potential regulatory functions requiring further studies (Finkel et al., 2021). Apart from experimental approaches, the availability of bioinformatic tools provided researchers with putative information to dissect the biology of SARS-CoV-2 through the prediction of coding sequences, protein domains and functions (Rao et al., 2014). The identification of gene products remains a challenging endeavor due to the disadvantages present in each method. For example, protein expression profiles could vary depending on the cell line used, temporal expression of genes, or incidental translation events. Bioinformatic approaches typically employ homology-based predictions, which could miss out lineage-specific accessory proteins. A combination of both experimental and computational methods could complement the shortfalls of each method and provide a more accurate profile. A recent comparative genomics approach identified 7 accessory proteins that are translated into conserved functional proteins, supported by datasets from experimental approaches such as proteomics, RNA sequencing, and ribosome profiling (Jungreis et al., 2021). This gives rise to a reference set consisting of functional protein-coding genes: ORF1a, ORF1ab, S, ORF3a, ORF3c, E, M, ORF6, ORF7a, ORF7b, ORF8, N, and ORF9b. Further experiments, such as Western Blot, may aid in the discovery of viral protein expression. For example, the controversial ORF10 was recently found to be expressed in a clinical isolate using anti-ORF10 antibodies generated from sheep (Pancer et al., 2020; Rihn et al., 2021).

### Overexpression Studies

As with all newly emerging pathogen, our understanding of how SARS-CoV-2 evades the immune system, and the functional roles of its genes are limited. Gene overexpression studies are commonly performed to further characterize genes of unknown functions (Prelich, 2012). In such studies, a gene of interest (typically cloned into a plasmid) is introduced into a host system to investigate the phenotypic effects and elucidate its functional role. Using knowledge gained from our understanding of how SARS-CoV proteins inhibit IFN induction and signaling, several candidates present in SARS-CoV-2 have been investigated and shown to also exhibit anti-IFN properties (Sa Ribero et al., 2020).

Several studies have utilized IFNβ-promoter firefly luciferase reporter assay as a readout of IFN suppression to screen for IFN antagonists. A study by Yuen et al. (2020) introduced a panel of expression plasmids consisting of 27 SARS-CoV-2 proteins in HEK293FT cells and found that ORF6 strongly antagonized IFN expression, with NSP13, NSP14 and NSP15 exhibiting similar activities; Lei et al. (2020) screened 23 proteins and identified M, NSP1, NSP3, NSP12, NSP13, NSP14, ORF3, and ORF6 inhibited IFNβ activation; Li et al. (2020) screened a total of 9 structural and accessory genes and identified ORF6, ORF8, and N as IFN inhibitors; Vazquez et al. (2021) screened 10 of the 16 NSPs and 4 (NSP1, NSP11, NSP13 and NSP14) were shown to inhibit IFN production. Targeted overexpression studies have also suggested ORF3b and ORF9b to act as IFN antagonists (Jiang et al., 2020; Konno et al., 2020; Wu et al., 2021). Recently, the controversial ORF10 was shown to suppress type I IFN production when overexpressed in HeLa-ACE2 cells (Li X. et al., 2021). The authors also co-expressed proteins in the RIG-I/MAVS signaling pathway and found that overexpression of ORF10 resulted in a significant decrease in the levels of MAVS, suggesting that it interferes with IFN induction through the MAVS pathway. This study highlights how overexpression studies could provide mechanistic insight of a novel protein through co-expressing host factors in a suspected pathway that it affects. With the increasing detection of SARS-CoV-2 variants, mutagenesis of single genes could also be performed to identify how the mutations affect IFN levels and inform surveillance for variants that are more capable of evading the innate immune response.

Taken together, the overexpression of at least NSP1, NSP3, NSP11, NSP12, NSP13, NSP14, NSP15, ORF3, ORF6, ORF8, ORF9b, ORF10, N, and M have been implicated to antagonize IFN induction, though these results were not consistent across studies. In a study by Li A. et al. (2021) further experiments showed that NSP12 is not a IFNβ antagonist although IFNβ promoter assays showed otherwise. The authors suggested that different experimental setups, plasmid backbones and fusion tags could have affected the luciferase readings and provided false positive results. In addition, while the overexpression of ORF8 was found to inhibit type I IFN, a study using an isolate lacking ORF8 showed similar IFN expression with the wild-type strain (instead of higher IFN expression), suggesting that ORF8 was dispensable and functional redundancies may exist between the multiple SARS-CoV-2 proteins (Gamage et al., 2020). Thus, it is important that findings from overexpression studies be validated (such as using an infectious clone), and combinations with other techniques (e.g., protein or RNA interaction studies) are needed to gain further insights into the pathway and mechanism through which IFN is suppressed, as well as the relative contribution of individual viral proteins in antagonizing IFN activity. Furthermore, results should be interpreted cautiously as these viral proteins may not reach such high levels as to cause an effect in the host during normal infection.

It is expected that more proteins could play a role in viral innate immune evasion owing to the large genome size of SARS-CoV-2 and the presence of numerous non-canonical transcripts with unknown function. Overexpression, while with its caveats, is a relatively simple and quick approach as a first line of screening to search for viral antagonists for antiviral factors.

### Protein-Protein Interaction Studies

While studies utilizing overexpression could help to tease out viral proteins involved in innate immune evasion (e.g., general reduction in IFNs or ISGs), protein-protein interaction (PPI) studies are necessary to determine the host factor involved and the pathway affected. Comprehensive PPI maps using AP-MS have been performed by Gordon et al. (2020a,b) and various innate immune signaling proteins have been identified to be targeted by SARS-CoV-2 proteins. For example, NSP13 was found to interact with TBK1 and TBKBP1 of the IFN pathway; NSP15 interacts with RNF41 (an activator of TBK1 and IRF3). The authors also identified interactions between SARS-CoV-2 ORF6 and the NUP98–RAE1 complex (nuclear export factors), consistent with a later finding by Miorin et al. (2020) which found that this interaction affected STAT nuclear import and IFN-I signaling. The interaction of ORF9b with TOM70 (mediates activation of IRF3) was also found by Jiang et al. (2020) using co-immunoprecipitation, and further experiments showed that this inhibited IFN-I responses. A recent mechanistic analysis of ORF9b by Han et al. (2021) using co-immunoprecipitation found that ORF9b inhibited the RIG-I/MDA-5–MAVS, TLR3–TRIF, and cGAS–STING signaling pathways. The structural protein, N, has been found to interact with STAT1, STAT2, TRIM25, RIG-I (Mu et al., 2020; Gori Savellini et al., 2021; Oh and Shin, 2021), while M interacts with MDA5, TRAF3, IKKϵ, and TBK1 (Fu et al., 2021; Sui et al., 2021) to attenuate the innate immune response. Furthermore, SARS-CoV-2 M protein has also been found to promote TBK1 degradation *via* promoting K48 ubiquitination (Sui et al., 2021). The SARS-CoV-2 NSP3 (papain-like protease) preferentially cleaves ISGylated (ISG15) substrates, leading to reduced IRF3/TBK1/P65 phosphorylation and reduced IFN activation (Frieman et al., 2009; Shin et al., 2020). An important consideration to note is that these studies were performed using overexpressed proteins, often in the context of single viral proteins. To better recapitulate the interactions during viral infection, viral-infected cell lysates may be used for pull-down experiments. In this way, interactions requiring multiple indirect viral protein partners may also be identified.

### RNA-RNA Interaction Studies

As an RNA virus with a relatively large genome (∼30 kb) and multiple subgenomic RNAs, it is expected that various RNA-RNA interactions may exist between the virus and host. Indeed, a comprehensive mapping of RNA-RNA interactions in infected Vero E6 cells revealed ∼300 host RNAs that interact with the SARS-CoV-2 genomes, with mitochondrial RNAs and snoRNAs being strong interactors (Yang et al., 2021). The authors suggested that the binding with snoRNAs recruit 2′-O-methylation modifications which may aid in evasion of host innate immune recognition (Dimitrova et al., 2019). However, as Vero E6 cells are IFN induction-deficient, many possible interactions with the RNAs of host innate immune molecules may have been missed.

## Recommendation of Approach for the Identification and Elucidation of Host-Viral Interactions Involved in Viral Evasion Strategies

Current methods of investigating host-viral interactions rely greatly on overexpression, such as luciferase reporter screens or single protein overexpression followed by pull-down to identify PPIs. As discussed in the previous section, the overexpression of viral proteins may lead to false positive findings due to the artificially elevated levels of viral proteins not found in normal infection. Viruses, utilizing their repertoire of DNA/RNA and proteins from their small genome, adapted multiple means of subverting the innate immune response to ensure their replication at the detriment of the host. The identification of these mechanisms is important to devise strategies to dampen the virulence of a virus and prevent severe infection and loss of life during an outbreak. Thus, future studies should move toward the use of whole virus and biologically relevant cell systems to elucidate the multipronged strategies employed by viruses to evade the innate immune system.

### Reverse Genetics

Reverse genetics (RG) is a powerful method which allows the study of viral proteins in the whole virus context which more accurately reflects the virus-host interactions during normal infection. The method utilizes an infectious clone generated from full-length cDNA of the virus and allows mutagenesis to be performed to investigate the functional roles of certain genes of interest. Many groups have developed RG systems for SARS-CoV-2, with some safe for use in Biosafety Level 2 laboratories, opening SARS-CoV-2 related studies to more researchers (Thi Nhu Thao et al., 2020; Xie et al., 2020; Ju et al., 2021; Nguyen et al., 2021; Rihn et al., 2021). However, due to the difficulties of generating RG systems, there is a lack of publication utilizing RG to investigate the anti-IFN activity of individual SARS-CoV-2 proteins. In a recent study, the authors used a recombinant SARS-CoV carrying ORF6 from SARS-CoV-2 and found that SARS-CoV-2 ORF6 was less potent than SARS-CoV ORF6 in inhibiting the innate immune response (Schroeder et al., 2021). However, it remains to be seen whether this is reproducible in the SARS-CoV-2 context as there could be SARS-CoV-2 specific proteins that may amplify the effects of SARS-CoV-2 ORF6.

### Choosing an Appropriate Host System

For the detection of host-viral interactions that can lead to immune evasion, a crucial component of the investigation is the selection of an appropriate host system to reconcile the findings and its biological relevance. Vero E6 is commonly chosen as a host system as it is highly susceptible to SARS-CoV-2 infection. However, the disadvantages of using Vero E6 to study host cellular response are that (i) it originates from monkeys, and (ii) it is known to lack type I IFN genes (though downstream ISGs may be induced by externally introduced IFN), which makes it difficult to resolve the complexity of host innate immune responses to SARS-CoV-2 in humans (Emeny and Morgan, 1979; Saccon et al., 2021). Other cells lines for investigating the innate immune responses to SARS-CoV-2 have been employed, such as Caco-2 (human intestinal epithelium) and Calu-3 (human lung epithelium) (Shuai et al., 2020; Saccon et al., 2021). In relation to respiratory viruses, the nasal epithelium is thought to be the first site of viral contact for viral entry into the host and is equipped with elements of innate immunity (e.g., mucociliary barrier and type I/III innate immune responses), serving early antiviral response functions (Gallo et al., 2021). The use of differentiated human nasal epithelial cells (*ex vivo*) grown in 3D air-liquid interface cultures have been shown to exhibit the anatomical and physiological processes found *in vivo* (Zhao et al., 2012) and could serve as a versatile research tool in the study of respiratory viral-host interactions. Other *ex vivo* cultures with SARS-CoV-2 tropism include the human conjunctiva, bronchial and lung tissues (Hui et al., 2020). As primary cell lines, these models could also allow the study of biological variability (Yan et al., 2016). Recent advances in 3D cultures have facilitated the development of organoids which better mimic the complexity of human physiological processes. For example, studies have shown infection of SARS-CoV-2 in airway or lung organoids greatly contribute to the understanding of viral pathogenesis in the respiratory tract and identify cellular tropisms (Gamage et al., 2020; Vanderheiden et al., 2020; Mallapaty, 2021; Mulay et al., 2021). Furthermore, single cell analyses could be carried out to identify changes in different cell types (Gamage et al., 2022). This can also be extended further to organoids from other organs to recapitulate systemic or organ tropism of SARS-CoV-2 and other novel viruses (Giobbe et al., 2021; Troisi et al., 2021).

### Protein-Protein Interactions

In terms of human viral infection, protein-protein interaction between viral and host proteins remains the mainstay of where we identify viral innate immune evasion mechanisms. To study the large permutation of interaction between viral and host proteins, high-throughput methods followed by targeted validation are the main methods widely used to elucidate such interactions.

*In silico* methods are mostly based on the mRNA sequence, structural similarity, and phylogeny and can be used to predict interaction between viral proteins and host proteins These methods can be used to guide verification with *in vivo* and *in vitro* methods. *In vitro* methods can be further categorized into targeted and non-targeted methods. In non-targeted methods, a general screen is done to identify all possible interaction pairs in a set of proteins, which in the case of viral-host interaction studies can be performed on the cell lysate of infected cells. In targeted methods, a protein of interest (bait) is expressed and used as the focal point to identify all PPIs with that protein. *In vivo* methods mainly make use of two-hybrid systems in simple model organisms. Two-hybrid systems are usually based in yeast, where interaction between proteins (one bound to a DNA-binding domain and the other to an activation domain) recombinantly expressed in the yeast host is required for reporter gene expression (Fields and Song, 1989). *In vivo* methods and targeted methods are more suited for verification of the interactions identified in the general screen.

Targeted methods include co-immunoprecipitation, affinity purification (AP), tandem AP, and proximity labeling. Co-immunoprecipitation utilizes immobilized antibodies on beads/surfaces against a protein of interest (bait). The infected cell lysate/protein cocktail is then allowed to interact with the beads/surface, resulting in the binding of the bait to the immobilized antibody. With a robust interaction between the bait and any prey (proteins that are able to interact with the bait), the bait-prey complex will remain associated with the immobilized antibody when the non-specific proteins are washed off, leaving the bait-prey complex bound to the beads (Phizicky and Fields, 1995; Berggard et al., 2007; Iacobucci et al., 2021). Further procedures can then be performed (such as MS) to identify the interacting protein. AP is similar to co-immunoprecipitation; instead of immobilized antibodies, the bait protein is immobilized on the bead (Havis et al., 2017). Tandem AP has a few additional steps compared to AP. The bait protein is expressed with a tag that binds to the immobilized antibodies; the tag allows the bound protein complex to be cleaved from the beads after the non-specific proteins have been removed by a wash step. A different set of beads that bind the newly exposed epitope on the cleaved tag is then introduced, allowing a second wash step that increases the purity and accuracy of the results (Rohila et al., 2004; Adelmant et al., 2019). The bound complex can then be removed from the beads by an eluent for further procedures to identify the interacting proteins.

Proximity dependent labeling is based on labeling proteins in close proximity to the protein of interest. This method is useful for determining transient interactions in the protein interactome, as the extensive washing steps required to eliminate non-specific proteins for AP and co-immunoprecipitation may result in the destabilization of such transient interactions. BioID, the first proximity labeling strategy, is based on BirA, a biotin ligase that has been adapted to biotinylate proteins in close proximity (∼10 nm) upon exposure to biotin and ATP (Roux et al., 2013). The biotin ligase is fused to the protein of interest and biotinylated proteins can then be isolated with streptavidin beads and analyzed with further procedures.

Non-targeted methods include crosslinking investigations, which are based on the premise of forming covalent bonds between amino acid residues that are in proximity. The parameters of the crosslink (positions, types, length, number) depend heavily on the crosslinker used, with each having its own set of advantages and disadvantages. Crosslinking is followed by purification of crosslinked proteins and finally identification by MS (Matzinger and Mechtler, 2021).

### RNA-RNA Interactions

While viral-host RNA-RNA interaction is uncommon in mammalian viruses, the advent of RNA-RNA interaction platforms can serve to uncover novel interaction at the RNA levels between virus and host. For example, studies of small RNA plant viruses have shed light on RNA silencing (Wang et al., 2012). Much work remains to be done on understanding the RNA-RNA interactions between the human host and viruses, which may yield novel innate immune evasion mechanisms.

For the elucidation of transcriptome wide RNA-RNA interactions, which would be ideal for identifying any interactions between viral RNA with the host RNA, CLASH (crosslinking, ligation, and sequencing of hybrids) (Kudla et al., 2011; Helwak et al., 2013), PARIS (psolaren analysis of RNA interactions and structures) (Lu et al., 2016, 2018), SPLASH (sequencing of psolaren crosslinked, ligated and selected hybrids) (Aw et al., 2016, 2017), LIGR-seq (ligation of interacting RNA followed by high-throughput sequencing) (Sharma et al., 2016), MARIO (Mapping RNA interactome *in vivo*) (Nguyen et al., 2016) and RIC-seq (RNA *in situ* conformation sequencing) (Cai et al., 2020) are some of the most common methods. These methods can be performed on *in vitro* or *in vivo* samples.

These methods follow a general framework. Psolaren or one of its derivatives, AMT, is used to crosslink interacting RNAs. In context, infected cell lysate/an infected cell can be used to pinpoint viral-host RNA interactions. This is followed by a purification step, such as gel purification, to isolate the crosslinked interacting RNA molecules. Each individual interacting RNA duplex is then subjected to proximity ligation to ensure that their interaction is preserved. Interacting complexes are then isolated before the crosslink is reversed, releasing both RNAs (though they remain associated due to proximity ligation). This preserved interaction allows sequencing methods, such as PORE-cupine (RNA structure analysis using nanopore sequencing) (Aw et al., 2021), to pinpoint the location and the secondary structures of the interacting RNA fragments on the viral and host genome. This would allow an accurate and near complete representation of the viral-host RNA-RNA interactions. Once the viral-host RNA interactome has been determined, specific RNA-RNA interactions can be validated by methods such as surface plasmon resonance (SPR) (Di Primo et al., 2011), electrophoretic mobility shift assay (EMSA) (Bak et al., 2015), or Förster resonance energy transfer (FRET) (Hardin et al., 2015).

### RNA-Protein Interactions

These methods are applicable to both Viral (Protein)—Host (RNA) and Viral (RNA)—Host (Protein) interactions. The methods may be divided into RNA-targeted (using RNA as bait) and protein-targeted (using protein as bait).

For RNA-targeted methods, they share a commonality in that a tag is expressed on in vitro transcribed RNA, or a probe is bound to the RNA of interest. This tag, whether biotin (Zheng et al., 2016) or an aptamer (Hartmuth et al., 2004; Faoro and Ataide, 2014), is used to bind streptavidin beads. Infected cell lysate can then be introduced to facilitate formation of RNA-protein complexes. Non-specific proteins can then be removed with wash steps, leaving the RNA-protein complexes of interest behind for further analysis. Alternatively, fluorophore-bound recombinant viral RNA of interest can be added to a protein microarray to allow formation of RNA-protein complexes. Fluorescence can then be used to determine the proteins that are interacting with the RNA (Kretz et al., 2013). This has been done with a protein microarray consisting of 9,400 recombinant human proteins (Human ProtoArray) (Kretz et al., 2013).

These methods can be further differentiated by the need for a crosslinker. Crosslinkers commonly used in RNA-protein interaction studies are UV light (Li et al., 2014) and formaldehyde (Vasudevan and Steitz, 2007). Each crosslinker has their own specificities and properties, and care must be taken to decide upon the correct crosslinker to use (Li et al., 2014).

A range of methods are available for exploitation: RAP (RNA antisense purification) (Hacisuleyman et al., 2014), PAIR (peptide-nucleic-acid-assisted-identification of RNA binding proteins) (Zeng et al., 2006), MS2-BioTRAP (MS2 *in vivo* biotin-tagged RAP) (Tsai et al., 2011), TRIP (tandem RNA isolation procedure) (Matia-Gonzalez et al., 2017), ChIRP (chromatin isolation by RNA purification) (Chu et al., 2011), CHART (capture hybridization analysis of RNA targets) (Simon et al., 2011) and VIR-CLASP (viral crosslinking and solid-phase purification) (Kim et al., 2021).

One method that does not require crosslinking makes use of proximity labeling instead. RaPID (RNA-protein interaction detection) (Ramanathan et al., 2018) utilizes a mutant BirA to biotinylate proteins interacting with recombinant BoxB flanked RNA of interest. Biotinylated proteins can then be purified with streptavidin beads (Ramanathan et al., 2018).

For protein-targeted methods, CLIP (crosslinking and immunoprecipitation) can be utilized (Ule et al., 2003). Interacting RNA-protein complexes are stabilized with crosslinking [UV (Ule et al., 2003) or otherwise (Kim and Kim, 2019)] before purification via immunoprecipitation of the protein of interest.

The purified complexes obtained can then be processed *via* high-throughput sequencing (Licatalosi et al., 2008) to identify the RNA and MS to identify the protein. With the identified interaction, further studies can look into verifying and functionally characterizing them.

### CRISPR Screens

CRISPR-Cas9 systems can be used to facilitate genomic screens by knocking out a single gene per cell from a library of genes that are suspected to be relevant to the viral infection/replication process. The resulting clone can then be expanded and subjected to viral infection. Cellular response and infection progression can then be monitored to determine if the gene contributes to viral infection. This information can then be used to perform targeted protein-protein interaction studies to further characterize the interaction (Koike-Yusa et al., 2014; Shalem et al., 2014; Poirier, 2017).

### Seeking out Innate Immune Impacting Interactions

Once the virus-host interactome has been determined, specific interactions can become the focus of the study. Interactions with host genes of interest related to the innate immune response (type I/III IFNs etc.) can be further investigated for impact on the innate immune response by perturbing and/or enhancing them. Pathogen sensors, IFN and ISG expressions are some of the metrics that can be used to approximate the impact of the interactions.

## Conclusion and Future Outlook

**FIGURE 1:**
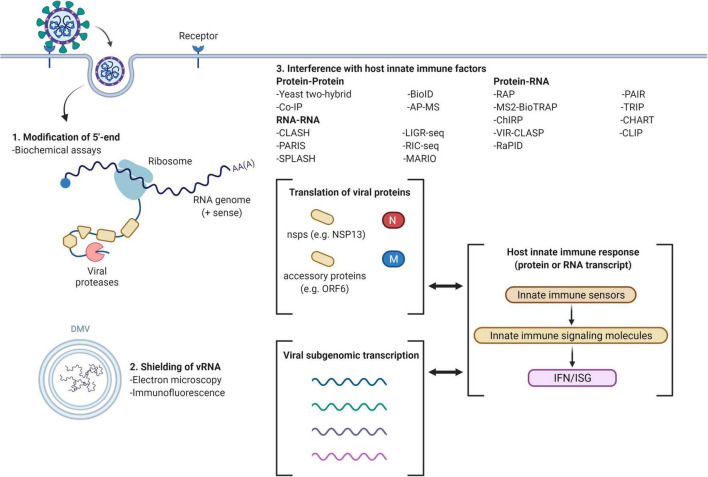
Viral evasion mechanisms and methods for investigation (example with SARS-CoV-2). Common methods of innate immune evasion by viruses include the modification of 5′-end, double membrane vesicles (DMV) to shield viral RNA (vRNA) and interference with host innate immune factors. 5′-end modifications may be determined using biochemical assays; formation of DMV can be detected by electron microscopy and immunofluorescence localization. The host innate immune response may be interrupted through protein-protein interactions, RNA-RNA interactions and viral (protein)-host (RNA)/viral (RNA)-host (protein) interactions, attenuating interferon (IFN) and interferon stimulated gene (ISG) expression. Figure created using BioRender.com.

In conclusion, the emergence of novel viruses like SARS-CoV-2 is an inevitability with the constantly expanding global population. The COVID-19 pandemic has demonstrated the lethality of such emerging pathogens which we have limited information about and the impact that they can have on our globalized society. Identifying viral evasion strategies is, thus, an important component of viral research to guide the development of therapeutics and halt transmission within the population, for both emerging and seasonal viruses. There are overlapping and common viral evasion strategies which provide a starting point to uncover these mechanisms and identify viral/host factors involved. From the studies reviewed in this paper, we see that the focus of the viral-host interactions studied are ones that have some form of homology to related viruses with known effect on the innate immunity. Furthermore, the focus seems fixated on overexpression studies (which is not representative of whole virus infection) and protein-protein interactions, with a lack of studies focusing on the RNA-RNA and RNA-protein interactions that emerging viruses may have. Here, by consolidating the current studies on SARS-CoV-2, a virus with strong antiviral evasion capabilities, we have identified areas that require integration of new approaches (e.g., a diversified focus on different classes of viral-host interactions, search for more novel interactions) to identify biologically relevant viral factors that can be targeted for management of viral infections (summarized in Table 1 and Figure 1).

**TABLE 1:** Methods to investigate interacting partners.

Method	Used with	Comments
**RNA-RNA**		
CLASH	SPR, EMSA, FRET	
PARIS	SPR, EMSA, FRET	
SPLASH	SPR, EMSA, FRET	
LIGR-seq	SPR, EMSA, FRET	
MARIO	SPR, EMSA, FRET	
RIC-seq	SPR, EMSA, FRET	
**Protein-Protein**		
Yeast two-hybrid		
Co-immunoprecipitation	MS	Requires antibody to viral protein which may not be readily available (especially for novel proteins)
(Tandem) Affinity purification	MS	
BioID	MS	Can be used for transient interactions
**Protein-RNA**		
RAP	RNA sequencing, MS	
PAIR	RNA sequencing, MS	
MS2-BioTRAP	RNA sequencing, MS	
TRIP	RNA sequencing, MS	
ChIRP	RNA sequencing, MS	
CHART	RNA sequencing, MS	
VIR-CLASP	RNA sequencing, MS	
CLIP	RNA sequencing, MS	
RaPID	RNA sequencing, MS	

## Author Contributions

DT, ZL, and KT contributed to the initial conceptualization of the manuscript and writing of the manuscript. DT and ZL contributed to literature search. DT, ZL, KT, and JC contributed to literature selection, review, and finalization of the manuscript. All authors contributed to the article and approved the submitted version.

## Conflict of Interest

The authors declare that the research was conducted in the absence of any commercial or financial relationships that could be construed as a potential conflict of interest.

## Publisher’s Note

All claims expressed in this article are solely those of the authors and do not necessarily represent those of their affiliated organizations, or those of the publisher, the editors and the reviewers. Any product that may be evaluated in this article, or claim that may be made by its manufacturer, is not guaranteed or endorsed by the publisher.
